# Undiagnosed hypertension in Croatia

**DOI:** 10.3325/cmj.2023.64.4

**Published:** 2023-02

**Authors:** Ana Ivičević Uhernik, Verica Kralj, Petra Čukelj, Ivana Brkić-Biloš, Marijan Erceg, Tomislav Benjak, Ranko Stevanović

**Affiliations:** Croatian Institute of Public Health, Zagreb, Croatia

## Abstract

**Aim:**

To determine the prevalence of undiagnosed hypertension in Croatia, and to assess its association with various demographic, socioeconomic, lifestyle, and health care usage factors.

**Methods:**

We used the data from European Health Interview Survey wave 3, conducted in Croatia in 2019. The representative sample consisted of 5461 individuals aged 15 years and older. The association of undiagnosed hypertension with various factors was assessed with simple and multiple logistic regression models. The factors that contribute to undiagnosed hypertension were identified by comparing undiagnosed hypertension with normotension in the first model and with diagnosed hypertension in the second model.

**Results:**

In the multiple logistic regression model, women and older age groups had lower adjusted odds ratio (OR) for undiagnosed hypertension than men and the youngest age group. Respondents living in the Adriatic region had a higher adjusted OR for undiagnosed hypertension than those living in the Continental region. Respondents who did not consult their family doctor in the previous 12 months and those who did not have their blood pressure measured by a health professional in the previous 12 months had a higher adjusted OR for undiagnosed hypertension.

**Conclusion:**

Undiagnosed hypertension was significantly associated with male sex, age from 35 to 74, overweight, lack of consultation with a family doctor, and living in the Adriatic region. The results of this study should be used to inform preventive public health measures and activities.

Hypertension is the main risk factor for all-cause mortality (1). As elevated blood pressure can damage the arteries, hypertension is the leading cause of cardiovascular disease. In 2019, the worldwide prevalence of hypertension in the age group 30-79 was 32% (626 million) among women and 34% (652 million) among men (2). The number of people with hypertension worldwide has doubled in the last 30 years due to changes in demographic trends and lifestyle. However, the age-standardized prevalence remains stable due to decreases in high-income countries and increases in some middle- and low-income countries (2).

Hypertension is a complex disease with many modifiable and unmodifiable risk factors, such as older age, family history, or low socioeconomic status (3). Behavioral risk factors include smoking, alcohol consumption, poor dietary habits, a high body mass index (BMI), salt intake, a sedentary lifestyle, and stress (4,5).

Asymptomatic hypertension, since it remains undiagnosed and untreated, is an important risk factor for cardiovascular diseases, disability, and premature death. Its estimated prevalence in Central and Eastern Europe is 23% among hypertensive women and 37% among hypertensive men; 17% of those with a diagnosis are not treated (2).

Hypertension is also an important public health challenge in Croatia. In a study conducted in 2003, based on the Croatian Adult Health Survey (CAHS), arterial hypertension prevalence was 40.5% in men and 34.9% in women (6). The EH-UH study (7) conducted in 2005 found the prevalence of arterial hypertension to be 35.5% in men and 39.7% in women.

Hypertension awareness in the EH-UH study was relatively high (72.6%) and above the European average. This percentage was higher in women and increased with age. A total of 59% of patients (above the European average) were treated with antihypertensive medications, and hypertension was controlled in 19.4% of patients. These findings are similar to the results of the CAHS (14.8%), and to the values reported in other European countries (7,8).

Data on the prevalence of undiagnosed hypertension in Croatia are scarce and outdated, and the only available study on the topic (7) assessed the association of undiagnosed hypertension with just a few socioeconomic factors. Therefore, the aim of this study is to determine the prevalence of undiagnosed hypertension in Croatia, and to assess its association with various factors. The results will be used to design public health interventions tailored to populations at increased risk of undiagnosed hypertension.

## Participants and methods

### Sample

This research used the data from the European Health Interview Survey (EHIS) wave 3, conducted by the Croatian Institute of Public Health together with health care centers and community nursing services, counties' institutes of public health, the Croatian Bureau of Statistics, and the Ministry of Health. A stratified random sample of private households was formed in two stages. For sampling, the population census from 2011 was used, corrected for persons who died and households that ceased to exist by autumn 2018. In the first stage, 400 segments (the primary sampling unit) with 1435 enumeration districts were selected, with each segment consisting of enumeration districts belonging to the same municipalities or a county. In the second stage, nine addresses (occupied dwellings) were randomly selected from each segment. The sample comprised households in 3600 dwellings, of which 2580 took part in this survey, with 5461 individuals aged 15 years and older. Respondents were interviewed by trained community nurses, mostly face-to-face or by telephone when face-to-face interviews were not possible. People who underwent a telephone interview (only 33 out of 5461) could still have their blood pressure measured because multiple contacts, both in person and via telephone, were allowed. The survey was conducted from April until December 2019.

Before the start of the interviews, all respondents signed informed consent (a parent or legal guardian signed for respondents under 18 years). Weighting was applied to ensure the representativeness of the results (9).

### Materials

Hypertension status was determined in two ways: on the basis of an interview question (“During the past 12 months, have you had any of the following diseases or conditions: high blood pressure?”; question code CD1E), and on the basis of the mean value of two blood pressure measurements. The measurements were made before and after the interview with at least 20 minutes’ interval between them. If the mean value for systolic blood pressure was 140 mm Hg or higher, and/or the mean value for diastolic blood pressure was 90 mm Hg or higher, the person was classified as being hypertensive (10).

The respondents were classified as having “undiagnosed hypertension” if they answered “no” to the CD1E question and if the mean blood pressure values were above the cut-off. They were classified as having “diagnosed hypertension” if they answered “yes” to the CD1E question, regardless of the results of the blood pressure measurements. They were classified as “normotensive” if they answered “no” to the CD1E question and if the mean blood pressure values were below the cut-off.

Age was classified into ten-year age groups, up to 75+, except for the groups 15-24 and 25-34 years, which were pooled together due to a low prevalence of both undiagnosed and diagnosed hypertension in the age group 15-24 years. Marital status was divided into four categories: never married, married/registered partnership, widowed, and divorced. Regions of residence were Adriatic or Continental, which were the only two NUTS-2 level regions in Croatia in 2019 (11). The degree of urbanization was classified in line with the Degree of Urbanization Classification of Local Administrative Units as follows (12): cities, towns and suburbs, and rural areas. For the purpose of this study, cities, towns, and suburbs were analyzed as a single category: urban areas. The education level was classified according to the International Standard Classification of Education from 2011 (13) into low (primary school or less), medium (secondary school), and high (bachelor or college degree). Household income quintiles were calculated on the basis of the number of household members, household structure, and its total net monthly income.

Self-reported height and weight were used to determine the body mass index (BMI). Body weight was classified as normal weight or underweight (BMI<25 kg/m^2^), overweight (25 kg/m^2^≤BMI<30 kg/m^2^), and obesity (BMI≥30 kg/m^2^). Respondents were classified into smokers and non-smokers, as well as into those who drank alcohol and those who did not drink. Respondents were also divided into those who reported a consultation with their family doctor in the previous 12 months and those who did not. The same was applied for blood pressure measurement by a health professional in the previous 12 months.

### Statistical analysis

All calculations were done on weighted data. The data are expressed as counts (percentages) and 95% confidence intervals (CI). The Pearson χ^2^ test was used to assess the significance of differences in frequencies between the groups. Logistic regression was used to explore the association of undiagnosed hypertension (dependent variable) with different predictor variables (independent variables) compared with normotension and with diagnosed hypertension. The association of each independent variable with undiagnosed hypertension was assessed with simple and multiple logistic regression (with backward stepwise selection). The analysis was performed with SPSS, version 27 (IBM Corp. Armonk, NY, USA).

## Results

Overall, 2963 (55.7%) respondents were normotensive (95% confidence interval [CI] 54.0-57.4), 2121 (37.2%) had diagnosed hypertension (95% CI 35.6-38.9), and 372 (7.1%) had undiagnosed hypertension (95% CI 6.2-8.0), ie, the prevalence of diagnosed hypertension was approximately 5 times higher than that of undiagnosed hypertension. The total prevalence of hypertension was 44.3%.

Significant differences among three groups were found in all the analyzed characteristics (*P* < 0.05) (Table 1). The prevalence of undiagnosed hypertension was higher among men than among women. It increased from the youngest age group to the 45-54 age group, was stable in the 55-64 and 65-74 age groups, and decreased in the 75+ age group compared with younger age groups. The prevalence of diagnosed hypertension increased from the youngest to the oldest age group, resulting in the percentage of diagnosed and undiagnosed hypertension being almost the same in the youngest age group. The prevalence of undiagnosed hypertension in the oldest age group was 13 times lower than that of diagnosed hypertension. Differences in the prevalence of diagnosed and undiagnosed hypertension according to other investigated characteristics are presented in Table 1.

**Table 1:** Differences between groups according to hypertension status

	Normotension			Diagnosed hypertension			Undiagnosed hypertension			Pearson χ2 test *P* value
Variables	n	%	95% CI*	n	%	95% CI*	n	%	95% CI*	
**Sex**										<0.001
male	1278	54.5	51.7-57.0	858	35.7	33.2-38.3	232	10.0	8.6-11.6	
female	1685	56.7	54.5-58.9	1263	38.4	36.3-40.6	140	4.9	4.0-6.0	
**Age (years)**										<0.001
15-34	981	96.0	94.1-97.2	23	1.8	1.1-2.9	17	2.3	1.3-4.0	
35-44	551	85.5	82.0-88.4	66	9.9	7.5-12.9	31	4.6	3.1-6.9	
45-54	492	63.8	59.3-68.0	225	26.4	22.6-30.6	79	9.8	7.5-12.7	
55-64	464	47.2	43.4-51.0	515	43.1	39.4-46.9	105	9.7	7.7-12.2	
65-74	248	27.3	23.7-31.1	585	62.5	58.4-66.5	83	10.2	7.8-13.4	
75+	225	21.7	18.8-24.9	707	72.6	69.1-75.8	57	5.7	4.2-7.6	
**Marital status**										<0.001
never married and never in a registered partnership	883	85.6	83.0-87.9	131	10.4	8.5-12.7	50	4.0	2.9-5.4	
married or in a registered partnership	1701	53.6	51.4-55.8	1287	38.8	36.7-41.0	234	7.6	6.5-8.9	
widowed or in registered partnership that ended with death of partner	207	23.6	20.3-27.3	614	68.7	64.6-72.5	57	7.7	5.5-10.8	
divorced or in registered partnership that was legally dissolved	133	56.4	48.1-64.3	79	33.0	25.5-41.4	26	10.7	6.9-16.2	
**Place of residence**										0.008
urban	1722	57.3	55.1-59.6	1148	36.1	33.9-38.3	205	6.6	5.6-7.8	
rural	1241	52.2	49.9-54.5	973	39.7	37.5-42.0	167	8.1	6.7-9.6	
**Region**										<0.001
Continental	1971	53.2	51.1-55.3	1576	40.2	38.1-42.2	253	6.6	5.7-7.7	
Adriatic	992	60.4	57.6-63.3	545	31.6	28.9-34.4	119	8.0	6.5-9.7	
**Education**										<0.001
primary or less	565	37.3	34.2-40.4	886	56.9	53.7-60.1	83	5.8	4.3-7.7	
secondary	1698	58.3	56.0-60.6	966	33.6	31.4-35.9	224	8.0	6.9-9.4	
higher than secondary	689	70.3	66.6-73.8	244	23.4	20.2-26.9	63	6.3	4.7-8.4	
**Net monthly equalized income of the household (quintiles)**										<0.001
1st (poorest)	276	42.4	37.9-47.0	353	49.6	44.9-54.2	44	8.0	5.4-11.8	
2nd	321	48.1	43.7-52.5	303	44.7	40.4-49.1	51	7.2	5.3-9.7	
3rd	339	52.7	47.9-57.5	277	39.9	35.3-44.7	58	7.4	5.4-9.9	
4th	373	56.6	51.7-61.5	248	35.0	30.5-39.8	51	8.3	6.0-11.5	
5th (richest)	434	64.0	59.3-68.5	191	28.8	24.6-33.3	50	7.2	5.2-10.0	
**Body mass index**										<0.001
<25 kg/m^2^(normal and underweight)	1323	74.5	72.0-76.8	417	20.0	17.9-22.2	98	5.6	4.4-7.0	
between 25 kg/m^2^ and 30 kg/m^2^ (overweight)	1041	50.4	47.6-53.2	859	41.0	38.3-43.8	164	8.6	7.1-10.3	
≥30 kg/m^2^ (obesity)	409	33.7	30.4-37.1	731	58.9	55.4-62.4	93	7.4	5.8-9.4	
**Smoking**										<0.001
no	2029	51.9	49.9-53.9	1729	41.1	39.2-43.1	270	7.0	6.0-8.1	
yes	928	66.6	63.6-69.5	387	25.9	23.3-28.8	102	7.4	5.9-9.3	
**Alcohol consumption**										<0.001
no	981	48.8	46.1-51.6	973	45.4	42.6-48.1	114	5.8	4.7-7.2	
yes	1802	59.6	57.3-61.8	1047	32.4	30.4-34.6	244	8.0	6.9-9.3	
**Consultation of a family doctor in the previous 12 months**										<0.001
yes	1946	48.6	46.6-50.6	1904	45.3	43.4-47.3	240	6.1	5.2-7.1	
no	994	75.7	72.6-78.6	213	14.3	12.1-16.9	130	10.0	8.1-12.2	
**Blood pressure measurement by a health professional in the previous 12 months**										<0.001
yes	1849	48.1	46.1-50.1	1837	45.2	43.2-47.2	241	6.7	5.7-7.8	
no	948	76.5	73.2-79.6	208	15.1	12.5-18.2	116	8.3	6.7-10.3	

*Confidence interval.

A simple logistic regression was applied to examine the association of different factors with undiagnosed hypertension compared with normotension. Sex, age, marital status, urban/rural residence, education level, and BMI were all significantly associated with undiagnosed hypertension compared with normotension (Table 2). However, when the influence of other factors was controlled for by the application of a multiple logistic regression model, factors that remained significantly associated with undiagnosed hypertension were sex, age, BMI, and consultation with the family doctor during the previous 12 months. Women had a lower adjusted odds ratio (OR) for undiagnosed hypertension compared with men. Age groups older than 45 had a higher adjusted OR for undiagnosed hypertension compared with the youngest age group (15-34 years). Overweight and obese respondents had a higher adjusted OR for undiagnosed hypertension compared with respondents with a normal weight. Respondents who did not consult their family doctor during the previous 12 months had a higher adjusted OR for undiagnosed hypertension compared with those who did (Table 2).

**Table 2:** Correlates of undiagnosed hypertension – results of a regression model*

	Undiagnosed hypertension vs normotension				Undiagnosed hypertension vs diagnosed hypertension			
Variable	OR unadjusted	95% CI†	OR multivariable-adjusted†	95% CI†	OR unadjusted	95% CI†	OR multivariable-adjusted†	95% CI†
**Sex**								
male	1.00		1.00		1.00			
female	0.47	0.35-0.62	0.50	0.37-0.68	0.46	0.34-0.61	0.58	0.42-0.80
**Age (years)**								
15-34	1.00				1.00			
35-44	2.30	1.12-4.74	1.90	0.91-4.00	0.37	0.15-0.92	0.55	0.21-1.43
45-54	6.54	3.39-12.62	6.12	3.12-12.01	0.29	0.13-0.67	0.45	0.19-1.11
55-64	8.76	4.61-16.66	8.36	4.22-16.56	0.18	0.08-0.40	0.25	0.10-0.60
65-74	15.94	8.14-31.23	14.97	7.26-30.90	0.13	0.06-0.29	0.21	0.09-0.51
75+	11.19	5.69-21.99	11.22	5.47-23.00	0.06	0.03-0.14	0.10	0.04-0.25
**Marital status**								
never married and never in a registered partnership	1.00				1.00			
married or in a registered partnership	3.05	2.12-4.38			0.51	0.34-0.77		
widowed or in registered partnership that ended with death of partner	7.05	4.25-11.68			0.29	0.17-0.50		
divorced or in registered partnership that was legally dissolved	4.07	2.26-7.34			0.85	0.44-1.64		
**Place of residence**								
urban	1.00				1.00			
rural	1.34	1.02-1.75			1.11	0.84-1.46		
**Region**								
Continental	1.00				1.00			
Adriatic	1.06	0.80-1.41			1.53	1.14-2.06	1.61	1.16-2.24
**Education**								
primary or less	1.00				1.00			
secondary	0.89	0.62-1.27			2.35	1.64-3.36		
higher than secondary	0.57	0.37-0.90			2.63	1.66-4.18		
**Net monthly equivalised income of the household (quintiles)**								
1st (poorest)	1.00				1.00			
2nd	0.80	0.46-1.39			1.00	0.58-1.74		
3rd	0.74	0.42-1.29			1.14	0.66-1.99		
4th	0.78	0.44-1.38			1.47	0.83-2.63		
5th (richest)	0.60	0.34-1.06			1.55	0.87-2.79		
**Body mass index**								
<25 kg/m^2^ (normal and underweight)	1.00				1.00			
between 25 kg/m^2^ and 30 kg/m^2^ (overweight)	2.29	1.65-3.17	1.47	1.03-2.12	0.75	0.53-1.06	0.63	0.43-0.94
≥30 kg/m^2^ (obese)	2.93	2.02-4.27	2.01	1.36-2.98	0.45	0.31-0.66	0.38	0.25-0.57
**Smoking**								
no	1.00				1.00			
yes	0.83	0.62-1.12			1.69	1.24-2.31		
**Alcohol consumption**								
no	1.00				1.00			
yes	1.12	0.84-1.51			1.92	1.42-2.58		
**Consultation of a family doctor in the previous 12 months**								
yes	1.00				1.00			
no	1.05	0.79-1.41	1.42	1.03-1.95	5.20	3.74-7.23	3.49	2.38-5.12
**Blood pressure measurement by a health professional in the previous 12 months**								
yes	1.00				1.00			
no	0.79	0.59-1.05			3.74	2.63-5.30	1.91	1.28-2.84

*Abbreviations: OR – odds ratio; CI – confidence Interval.

†backward stepwise logistic regression model was applied; initially all variables were included in the model; at each step a variable with the weakest relationship with undiagnosed hypertension was removed (empty cells refer to such variables), until only variables significantly related to undiagnosed hypertension remained in the model.

The factors associated with undiagnosed compared with diagnosed hypertension were also identified in a separate regression model. In a simple logistic regression analysis, undiagnosed, compared with diagnosed hypertension was significantly associated with sex, age, marital status, region, education level, BMI, smoking status, alcohol consumption, consultation with the family doctor in the previous 12 months, and blood pressure measurement by a health professional in the previous 12 months (Table 2). When controlled for the influence of other variables through a multiple logistic regression model, undiagnosed compared with diagnosed hypertension remained significantly associated with sex, age, region, BMI, consultation with the family doctor in the previous 12 months, and blood pressure measurement by a health professional in the previous 12 months. Women and older age groups had a lower adjusted OR for undiagnosed hypertension than men and the youngest age group. Respondents living in the Adriatic region had a higher adjusted OR for undiagnosed hypertension than those living in the Continental region. Overweight and obese respondents had a lower adjusted OR for undiagnosed hypertension compared with those with a normal BMI. Respondents who did not consult their family doctor in the previous 12 months and those who did not have their blood pressure measured by a health professional in the previous 12 months had a higher adjusted OR for undiagnosed hypertension (Table 2).

## Discussion

This study shows a high hypertension prevalence in Croatia, with a significant, although smaller than expected, proportion of undiagnosed hypertension.

The total prevalence of hypertension in the adult population of Croatia is above the average of the EU27 (14), and it is especially high when compared with populations outside of Europe (2). This points to hypertension as a public health problem in Croatia and to the need for programs addressing this issue. The estimated age-standardized hypertension prevalence of 27.8% in Croatia is similar to the values in other Central European countries (15).

Undiagnosed hypertension is an additional problem, as individuals who are unaware of their hypertension do not get treatment. According to our study, approximately one-sixth of all hypertensive patients in Croatia is undiagnosed. This is significantly less than what was reported both in other countries (2) and previously in Croatia (8). However, due to a high prevalence of both diagnosed and undiagnosed hypertension in Croatia, the number of undiagnosed patients remains considerable and should be recognized as a public health priority.

Our study found twice as many men as women with undiagnosed hypertension, a finding that is in line with other studies (2). Respondents aged 45-75 years had the highest odds of undiagnosed hypertension. Every tenth person in this age group had undiagnosed hypertension, which is double the odds among people above 75 years of age, and five times higher than the odds in the age group 15-34. A greater prevalence of undiagnosed hypertension was found among respondents who were overweight compared with those with normal weight. This highlights the importance of regular blood pressure control among the overweight population. Finally, respondents who consulted their family doctor in the last 12 months had a significantly lower prevalence of undiagnosed hypertension, which emphasizes the importance of reaching those who do not regularly use health care.

Simple and multiple logistic regression models showed that the factors that contribute to undiagnosed hypertension when compared both with normotension and diagnosed hypertension were similar, although there were some differences.

Both in a single and multiple regression model (controlled for other variables), and compared with both normotension and diagnosed hypertension, undiagnosed hypertension was significantly associated with sex, age, BMI, and a consultation with the family doctor in the previous 12 months. Another study identified undiagnosed hypertension to be significantly associated with sex, age, and overweight/obesity (16). However, in our study the elderly and overweight had a higher odds of having undiagnosed hypertension in the model when undiagnosed hypertension was compared with normotension and lower odds in the model when it was compared with diagnosed hypertension. These results indicate that a younger person with a normal BMI has a higher chance of being undiagnosed with hypertension, which should be considered when planning preventive activities. This finding could be explained by both physicians and patients being more aware of a possible hypertension risk in the elderly and overweight, as these are known risk factors for hypertension. However, as young people and those with a normal BMI can also have hypertension, everyone, regardless of their age and BMI, should regularly check their blood pressure.

Surprisingly, the residence in the Adriatic region of Croatia was associated with a higher odds for having undiagnosed hypertension than the residence in the Continental region (when compared with diagnosed hypertension, and after controlling for other factors). This finding emphasizes the need for improving the diagnostics of hypertension in the Adriatic region. Population in the Adriatic region traditionally consumes a diet more closely following the principles of the Mediterranean diet (17), and has lower rates of cardiovascular disease mortality (18). Therefore, health workers in the Adriatic region may perceive their patients’ risk for hypertension to be lower and thus be less inclined to check their blood pressure.

The lack of consultation with the family doctor in the previous 12 months and the lack of blood pressure measurement by a health professional in the previous 12 months were associated with a higher odds for undiagnosed hypertension compared with diagnosed hypertension. This finding is expected (5) and clearly identifies regular preventive care as one of the major tools in reducing the prevalence of undiagnosed hypertension.

In Croatia, there is no organized hypertension screening. According to the Croatian Plan and Program of Healthcare Activities for 2020-2022, blood pressure should be measured at least every two years in people over 40 years of age, and in adult patients aged 18-65 who have known risk factors (smoking, abnormal BMI) during the patient’s visit to their family physician. In practice, whether the family doctor measures blood pressure during a visit depends on many factors: the reason for the visit, the physician’s motivation and workload, and the patient’s risk for hypertension as perceived by the physician. Younger people with a normal BMI are rightly perceived as having a lower risk for hypertension, and they visit their family physician less frequently, which drastically reduces their chances for having blood pressure measured. An encouraging fact is a significant increase in the proportion of Croatian population that had their blood pressure measured by a health professional in the previous 12 months: from 59.5% in EHIS 2014 (19), to 74.9% in EHIS 2019 (20).

In this study, undiagnosed hypertension was not associated with education or income level, while other studies reported a higher odds for undiagnosed hypertension among respondents with lower education level or lower income (16). A possible explanation for this finding is that in Croatia the quality of the health care system does not largely depend on the income or education level. Namely, Croatian health care is financed from the contributions deducted from the salaries of the employed, and is accessible to the large majority of the population regardless of their income.

There are several limitations to this study. We determined the hypertension status on the basis of blood pressure measurements and of self-reported previous diagnosis of hypertension, which may be prone to subjective recollection and interpretation. Also, no specific instructions were given to community nurses on whether to disclose the measurement result to the respondents, which could have influenced their recollection. On the other hand, blood pressure measurement in respondents' homes could have reduced the prevalence of “white coat hypertension.”

In Croatia, primary prevention is regularly carried out through public health campaigns on hypertension risk factors and on the importance of regular blood pressure measurements. Educating patients and health professionals on the significance of early detection, therapy, and control of hypertension could further decrease the proportion of undiagnosed hypertension. Asymptomatic adults, including those perceived to have a lower hypertension risk, should be more frequently screened. The screening should be carried out in addition to primary prevention measures affecting known risk factors for hypertension. All the aspects of this problem can only be covered only through a multisectoral approach including various stakeholders.
